# The Protective Effect of Heme Oxygenase-1 on Liver Injury Caused by DON-Induced Oxidative Stress and Cytotoxicity

**DOI:** 10.3390/toxins13100732

**Published:** 2021-10-17

**Authors:** Zitong Meng, Liangliang Wang, Yuxiao Liao, Zhao Peng, Dan Li, Xiaolei Zhou, Shuang Liu, Yanmei Li, Andreas K. Nüssler, Liegang Liu, Liping Hao, Wei Yang

**Affiliations:** 1 Department of Nutrition and Food Hygiene, Hubei Key Laboratory of Food Nutrition and Safety, Tongji Medical College, Huazhong University of Science and Technology, Hangkong Road 13, Wuhan 430030, China; ztmeng@hust.edu.cn (Z.M.); wangll20210820@163.com (L.W.); d201981406@hust.edu.cn (Y.L.); 2020508030@hust.edu.cn (Z.P.); lidan753@hust.edu.cn (D.L.); m202075394@hust.edu.cn (X.Z.); lgliu@mails.tjmu.edu.cn (L.L.); lphao@mails.tjmu.edu.cn (L.H.); 2 Department of Nutrition and Food Hygiene, MOE Key Lab of Environment and Health, School of Public Health, Tongji Medical College, Huazhong University of Science and Technology, Hangkong Road 13, Wuhan 430030, China; 3 Hubei Center for Disease Control and Prevention, 6 North Zhuodaoquan Road, Wuhan 430079, China; ls_hbcdc@163.com (S.L.); 13507116990@163.com (Y.L.); 4 Hubei Provincial Key Laboratory for Applied Toxicology, Hubei Center for Disease Control and Prevention, 6 North Zhuodaoquan Road, Wuhan, 430079, China; 5 Department of Traumatology, BG Trauma center, University of Tübingen, Schnarrenbergstr. 95, 72076 Tübingen, Germany; andreas.nuessler@med.uni-tuebingen.de

**Keywords:** deoxynivalenol, oxidative damage, DNA repair, HO-1, antioxidant enzyme, autophagy

## Abstract

Deoxynivalenol (DON) is a kind of Fusarium toxin that can cause a variety of toxic effects. Oxidative stress and DNA damage play a critical role in the toxicity of DON. However, previous studies focused more on acute toxicity in vivo/vitro models and lacked subchronic toxicity study in vivo. The potentially harmful effect of DON given at doses comparable to the daily human consumption in target organs, especially the liver, which is the main detoxification organ of DON, is also still not fully understood. Otherwise, Heme Oxygenase-1 (HO-1) has also reduced cell damage under the DON condition according to our previous study. Therefore, we used a rodent model that mimicked daily human exposure to DON and further explored its mechanism of toxic effects on liver tissue and Hepa 1–6 cell line. We also used adeno-associated virus (AAV)-modified HO-1 expressing by tail vein injection and constructed lentivirus-Hepa 1–6 cell line for mimicking HO-1 protective ability under the DON condition. The main results showed that both 30 d and 90 d exposures of DON could cause low-grade inflammatory infiltration around hepatic centrilobular veins. The reactive oxygen species (ROS) and 8-hydroxy-2 deoxyguanosine (8-OHdG) increased during DON exposure, indicating oxidation stress and DNA damage. Significantly, AAV-mediated liver-specific overexpression of HO-1 reduced DON-induced liver damage and indirectly protected the abilities of antioxidant enzyme/DNA damage repair system, while AAV-mediated silence of HO-1 produced the opposite effect. In addition, we found that overexpression of HO-1 could enhance autophagy and combined it with an antioxidant enzyme/DNA damage repair system to inhibit DON-induced hepatocyte damage. Altogether, these data suggest that HO-1 reduces the oxidative stress and DNA damage caused by DON sub-chronic exposure through maintaining DNA repair, antioxidant activity, as well as autophagy.

## 1. Introduction

Deoxynivalenol (DON) is one of the most widely distributed trichothecenes and it has various toxic effects on humans and animals [1,2,3]. Acute DON exposure can cause vomiting, diarrhea, and even shock-like death. However, such clinical intoxications are rare in humans, who are most frequently, exposed to low DON doses without developing acute symptoms. Long-term low-dose DON exposure may cause anorexia, growth retardation, and immunotoxicity [4]. Therefore, the adverse effects of long-term exposure to the human daily intake of DON cannot be completely ruled out. According to the 2011 World Health Organization report, the average daily intake of DON by humans is 0.2–14.5 μg/kg bw/day [5]. In some countries, children’s intake of DON exceeds 1.0 μg/kg bw/day, which is the provisional maximum tolerable daily intake (PMTDI, PDI) [6]. Therefore, long-term low-dose daily exposure to DON may have become a global threat to human health [7].

DON is mainly metabolized and detoxified in the liver. A previous study found that after 14 days of exposure to DON (28 mg/kg bw/day), the antioxidant enzyme activities in the liver of mice decreased [8]. Yu et al. also reported similar conclusions [9]. Oxidative stress is an important mechanism involved in the toxicity of DON [8]. Superoxide dismutase (SOD), catalase (CAT), and glutathione peroxidase (GPx) are essential components of the antioxidant system. These antioxidant enzymes cooperated to maintain the oxidation-antioxidant balance. However, as the dose and time of DON exposure increase, the redox balance may be disrupted. For example, 0.25 μg/mL DON can cause a decrease in total superoxide dismutase (T-SOD) activity and glutathione (GSH) levels and increase reactive oxygen species (ROS) and malondialdehyde (MDA) levels [10]. When the diet of mice was contaminated with DON, the activities of CAT and SOD in the liver decreased, while the content of MDA increased [11]. Meanwhile, many studies have shown that DON can cause excessive ROS and induce DNA damage. For example, after 6 h of exposure to 10 µM DON, DNA damage in mouse sperm was significantly increased, accompanied by a 2.5-fold increase in MDA content [12,13]. However, it is not clear whether low dose sub-chronic DON exposure influences the liver’s antioxidant enzyme activities, DNA damage, and DNA repair ability.

In addition, autophagy is a highly conserved process of eukaryotic cell recovery and plays a vital role in cell survival and maintenance. A previous study showed that DON induced cytotoxicity and apoptosis by inhibiting the PI_3_K-AKT-mTOR signaling pathway and activating autophagy [14]. Another study also found that the expression of Lamp2, LC3, and mTOR mRNA in pig oocytes was abnormal when exposed to DON [15], which indicated that DON treatment could induce autophagy/apoptosis in porcine oocytes. However, there are a few studies on the mechanism of DON-induced liver damage related to autophagy. Otherwise, as one of the phase II detoxification enzymes, heme Oxygenase-1 (HO-1) degrades heme into biliverdin, CO, and Fe^2+^ through bilirubin reductase, forming endogenous protective substances to regulate cell oxidation and apoptosis [16]. These evidences also indicated that HO-1 has a hepatoprotective effect. For example, the overexpression of HO-1 can maintain the integrity of liver tissue [17], reduce liver fibrosis [18], and delay the development of non-alcoholic fatty liver [19]. A previous study found that the chemical silencing of HO-1 by zinc protoporphyrin (ZnPP) can exacerbate inflammation, oxidative stress, hepatocyte apoptosis, and liver damage [20]. In addition, the HO-1 system can protect the liver from ischemia/reperfusion injury by enhancing autophagy [21,22]. Our previous study also observed that HO-1 could reduce DON-induced oxidative stress in human primary lymphocytes [23].

All in all, in this work, we aim to evaluate the impact of subchronic intoxication in mice liver with DON given at doses below the non-observed-adverse effect level (NOAEL), i.e., 100 μg/kg bw [24]. Combing with excellent previous studies, we used 25 μg/kg bw of DON [25] to explore the relationship with different expression patterns in antioxidant enzyme system, DNA repair system, and autophagy through modifying HO-1 expression under the DON condition. This study can provide a reference for the toxic effects of DON in liver tissue for future research and show possible potential strategies for future clinical treatment.

## 2. Results

### 2.1. HO-1 Can Reduce Liver Damage Caused by Low-Dose DON Exposure

#### 2.1.1. HO-1 Alleviated Liver Inflammation Induced by Low-Dose DON 

**Figure 1:**
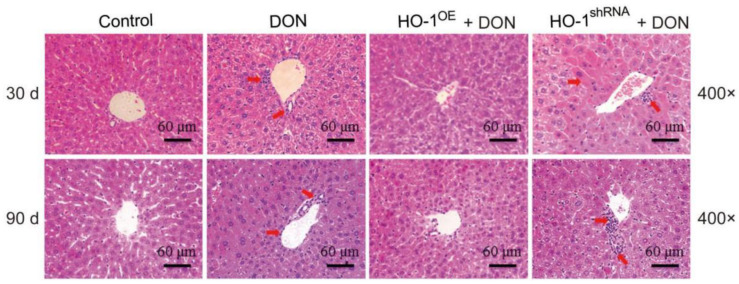
Hematoxylin-eosin (H&E) staining of mouse liver after 30 days and 90 days of Deoxynivalenol (DON) administration. The red arrow points to lymphocyte infiltration. The scale bar is 60 μm.

According to pathological sections, histological examination showed that 30-day treatment of DON triggered mild liver inflammation, which was reflected by the accumulation of lymphocytes around the hepatic centrilobular veins (Figure 1). To better mimicking human daily DON exposure, we prolonged DON administration to 90 days and observed a similar liver inflammatory response. In addition, compared with the DON group, the liver tissue damage in the HO-1^shRNA^ group was further aggravated. However, there was no obvious inflammatory reaction in the mouse liver of the HO-1^OE^ group (Figure 1). Collectively, these results indicated that sub-chronic low-dose DON exposure could trigger liver inflammation, the HO-1 could reduce the liver inflammation caused by DON. The number of lymphocytes in the same field of view (× 400) in one picture (N = 3) was calculated by Image J Software (National Institutes of Health, MD, USA).

#### 2.1.2. The Effects of DON Exposure Time and Dose on Hepa 1–6 Cell Viabilities

**Figure 2:**
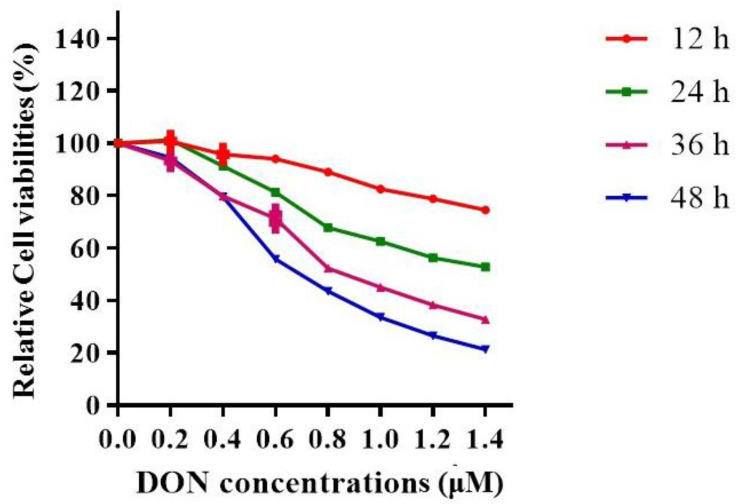
Cell counting kit-8 (CCK-8) measures the cytotoxicity of DON to Hepa 1–6. The cell survival rate of the control group was set to 100%. The test was performed three times independently, with three samples in each group.

To simulate the subchronic low-dose DON exposure in the cell, we used 0, 0.2, 0.4, 0.6, 0.8, 1.0, 1.2, or 1.4 μM DON to treat Hepa 1–6 cells for 12, 24, 36, and 48 h (Figure 2), and then tested the cytotoxicity by cell counting kit-8 (CCK-8). Based on the results, we selected 0.6 μM and 24 h as the treated concentration and exposure time respectively for subsequent cell intervention, to ensure enough cell viability and suitable cytotoxicity.

#### 2.1.3. HO-1 Inhibited the Levels of ROS and 8-OHdG Induced by DON

**Figure 3:**
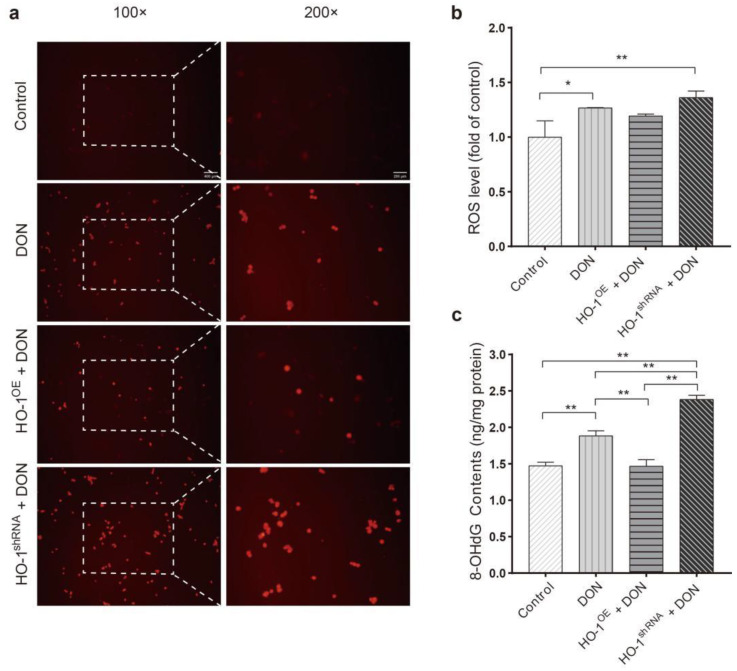
The levels of reactive oxygen species (ROS) and 8-hydroxy-2 deoxyguanosine (8-OHdG) in cells after DON administration. (**a**) Dihydroethidium (DHE) staining was photographed with an inverted fluorescence microscope, and the scale bar is 400 μm or 200 μm. (**b**) ROS levels in cells after exposure to DON. (**c**) 8-OHdG levels in cells after exposure to DON. Data are expressed as mean ± SD; “*” means *p* < 0.05; “**” means *p* < 0.01. The fluorescence intensity in the same field of view (×400) in one picture (N = 3) was calculated by Image J.

The levels of ROS and 8-OHdG were detected at the cellular level by DHE staining and ELISA, respectively. Compared to the control group, the DHE fluorescence intensity (Figure 3a,b) and 8-OHdG levels (Figure 3c) in the DON group and HO-1^shRNA^ group were significantly increased, and in the HO-1^OE^ group were significantly reduced. Overall, these results indicated that sub-chronic low-dose DON exposure could induce oxidative stress and DNA damage in Hepa 1–6, and HO-1 could reduce the above damages.

### 2.2. HO-1 Can Maintain the Antioxidant Enzyme/DNA Repair System in Mouse Liver during Low-Dose DON Exposure

#### 2.2.1. The Protective Effect of HO-1 after 30 Days of Exposure to DON

**Figure 4:**
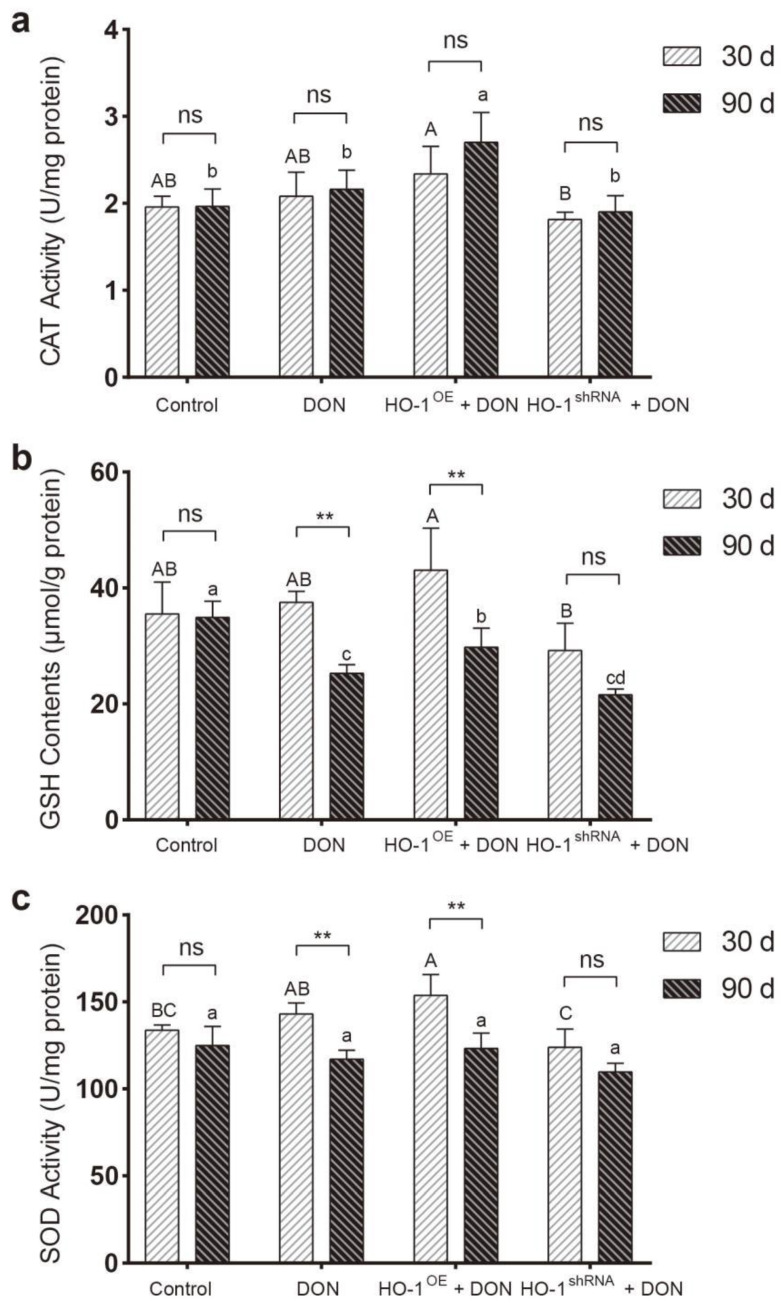
The effect of DON exposure for 30 days and 90 days on the activities of antioxidant enzymes in the liver of mice. (**a**) The activity of catalase (CAT) in the liver. (**b**) The content of glutathione (GSH) in the liver. (**c**) The activity of superoxide dismutase (SOD) in the liver. Data are means ± SD of three biological replicates. Different capital or lower-case letters indicated significant differences (*p* < 0.05) within the 90-day of DON administration groups or 30-day of DON administration groups, respectively. “**” means *p* < 0.01. The test was performed three times independently, with three samples in each group.

**Figure 5:**
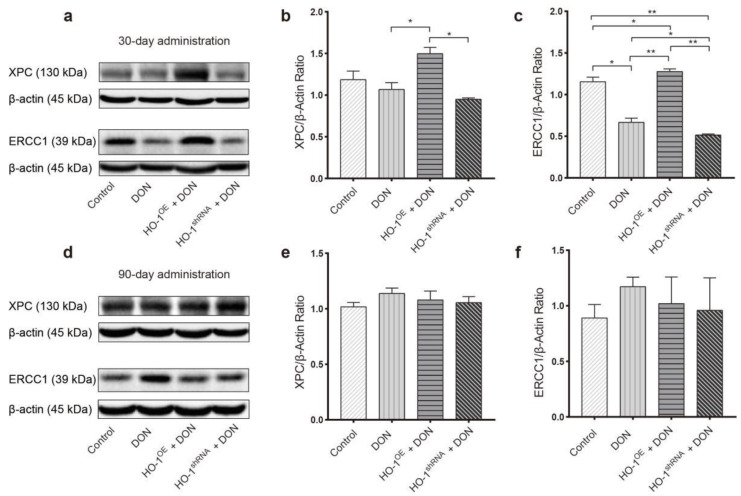
The effect of DON exposure on the expression of DNA repair protein in mouse liver. The expression levels of xeroderma pigmentosum complementation group C (XPC) and excision repair cross-complementation group 1 (ERCC1) in the liver after 30 (**a**–**c**) or 90 days (**d**–**f**) of exposure to DON. Data are expressed as mean ± SD; “*” means *p* < 0.05; “**” means *p* < 0.01. Western blot assays were performed three times independently and three samples per group.

After 30 days of exposure to DON, the content of GSH and the activities of CAT and SOD in the mouse liver of the HO-1^OE^ group were significantly higher than that of the HO-1^shRNA^ group (Figure 4). Compared to the DON group and the HO-1^shRNA^ group, the expression of excision repair cross-complementation group 1 (ERCC1) and xeroderma pigmentosum complementation group C (XPC) in the mouse liver of the HO-1^OE^ group was much increased (Figure 5a–c). These results revealed that HO-1 could activate the antioxidant enzyme system and enhance the DNA repair ability, after DON intervention for 30 days.

#### 2.2.2. The Protective Effect of HO-1 after 90 Days of Exposure to DON

After 90 days of exposure to DON, compared to the DON group and HO-1^shRNA^ group, both CAT activity and GSH content in the HO-1^OE^ group were significantly increased, despite the GSH contents in these three DON-treated groups were generally lower than the control group (Figure 4). Meanwhile, the expression levels of ERCC1 and XPC showed no significant difference among the four groups (Figure 5d–f). In addition, compared with 30 days of exposure to DON, the SOD activity and GSH content of the HO-1^OE^ group were largely reduced after DON intervention for 90 days (Figure 4). These results indicated that when the DON exposure time was extended from 30 days to 90 days, the redox system and the DNA repair system of the HO-1^OE^ group became imbalanced.

### 2.3. HO-1 Can Also Maintain the Antioxidant Enzyme/DNA Repair/Autophagy of Hepa 1–6 Cells during Low-Dose DON Exposure

#### 2.3.1. HO-1 Maintains Antioxidant Activity/DNA Repair in Hepa 1–6 Cells, under DON Exposure Conditions

**Figure 6:**
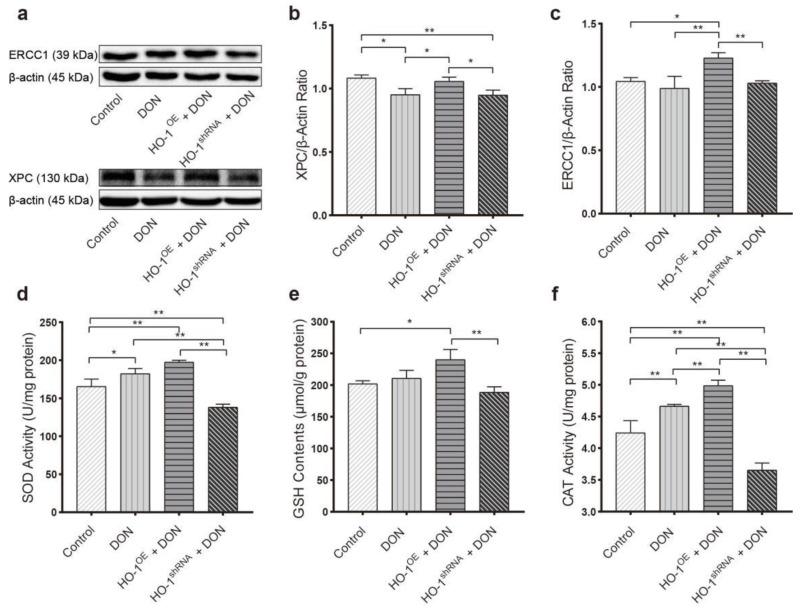
The effect of DON exposure on the expression of DNA repair protein and the activity of antioxidant enzymes in Hepa 1–6 cells. After 0.6 μM DON interfered with the cells for 24 h, (**a**–**c**) the SOD and CAT activities and the GSH content in the cells, (**d**–**f**) the expression levels of XPC and ERCC1 proteins in the cells. Data are expressed as mean ± SD; “*” means *p* < 0.05; “**” means *p* < 0.01. Western blot assays were performed three times independently and three samples per group.

We further explored the protective mechanisms of HO-1 in Hepa 1–6 cells under low-dose sub-chronic DON exposure. After the cells were exposed to 0.6 μM DON for 24 h, the HO-1^OE^ group presented a much higher content of GSH, and activities of SOD and CAT, compared to HO-1^shRNA^ groups and control groups (Figure 6a–c). Furthermore, the expression levels of ERCC1 and XPC in the HO-1^OE^ group were also significantly higher than the DON groups and HO-1^shRNA^ groups (Figure 6d–f). These results were similar to the animal experiment after 30-day DON exposure, which further confirmed that HO-1 could effectively activate the expressions of the antioxidant enzyme/DNA repair system in the early stage of sub-chronic DON exposure.

#### 2.3.2. HO-1 Could Protect the Ability of Autophagy in Hepa 1–6 Cells under DON Exposure Conditions

**Figure 7:**
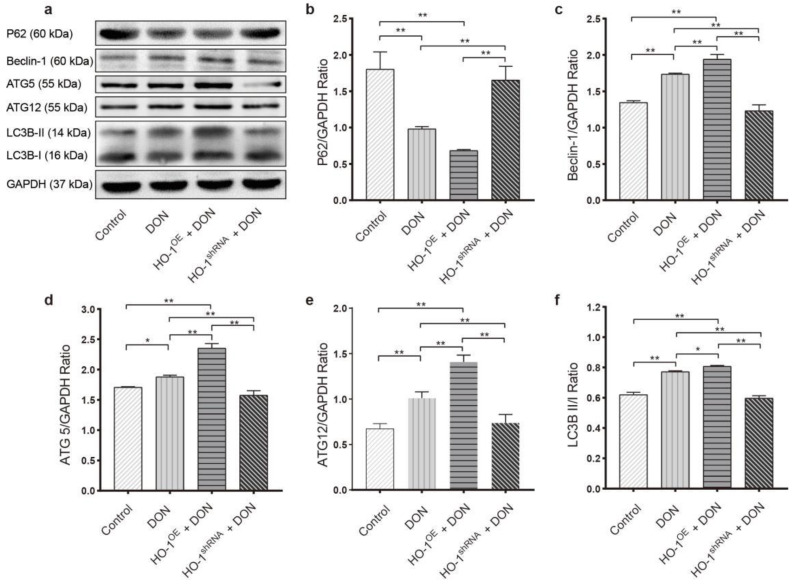
The effect of DON exposure on autophagy. (**a**–**f**) Detect autophagy-related proteins by Western blot: p62, LC3B, ATG5, ATG12, and Beclin-1. Data are expressed as mean ± SD; “*” means *p* < 0.05; “**” means *p* < 0.01. Western blot assays were performed three times independently and three samples per group.

Compared to the control, low dose of DON exposure led to increased expression levels of Beclin-1, ATG5, and ATG12, reduced expression levels of SQSTM1/p62, and decreased ratios of LC3B-I/LC3B-II (a free form of phosphatidylethanolamine to bound form), suggesting that the autophagy in Hepa 1–6 cells was significantly activated by the DON exposure (Figure 7). Moreover, these phenotypes can be greatly aggravated by the overexpression of HO-1 and can be significantly blocked by the silence of HO-1 (Figure 7). These results together indicated that HO-1 played an essential role in maintaining the autophagy ability within Hepa 1–6 cells.

## 3. Discussion

The liver is the main metabolism and detoxification organ of DON. Nevertheless, many previous studies of DON-induced hepatotoxicity have demonstrated inconsistent conclusions. For instance, previous studies have found that DON could induce the loss of ribosomes in hepatocytes and liver tissues of pigs [26,27]. DON treatment (25 μg kg^−1^) could cause piecemeal necrosis at the lobular-portal interface in the liver tissues of mice [25]. However, DON treatment (0.071 or 0.355 mg kg^−1^) did not cause any changes in their liver weights [28]. No significant lesions in the liver of pigs were observed under similar concentration [29]. In addition, accumulating evidence indicates that oxidative damage is one of the main mechanisms of DON inducing cytotoxicity, DNA damage, and apoptosis [30]. Meanwhile, many studies have also shown that ROS production in hepatocytes is related to the dosage and duration of DON administration. For instance, DON (0.1 μg/mL) could induce oxidative stress in rat liver clone 9 cells [31]. In addition, ROS participated in oxidative stress and acted as a mediator to induce DNA damage in HepG2 cells [32]. However, when HepG2 cells were incubated with DON (concentrations from 15 μM to 60 μM) for 1 h, the intracellular ROS levels did not increase significantly at lower concentrations but increased significantly (1.4 times) at 60 μM [32,33]. In conclusion, it is reasonable to understand that DON-dependent production of ROS is largely related to the hepatotoxicity induced by DON. Therefore, we take oxidative damage as the key point to explore the toxic effects of low-dose sub-chronic DON exposure on the liver. This study illustrated our speculation that sub-chronic low-dose DON exposure can cause pathological changes in the liver and induce Hepa 1–6 to produce excessive ROS.

The modifications in antioxidant enzyme activities have been used as a biosensor for ROS formation during oxidative stress in the cell system [34]. The role of SOD is the first line of defense of the biological antioxidant system. It can precisely remove superoxide anion free radicals generated in oxidative metabolism and convert them into O_2_ and H_2_O_2_ [35,36]. CAT is a terminal oxidase abundant in the mammalian liver and is an essential part of liver mitochondrial antioxidant reaction [37], its function is to catalyze H_2_O_2_ and decompose it into O2 and H_2_O to prevent over-oxidation. GSH-PX is an important peroxide decomposition enzyme, which catalyzes GSH (reduced glutathione) to GSSG (oxidized glutathione), decomposes H_2_O_2_ into H_2_O and O_2_ to reduce or eliminate the damage caused by peroxide [38] and it is the primary source of GSH in the liver [39]. Therefore, we evaluate the oxidative damage of the liver through the changes of antioxidant enzymes in liver tissue and Hepa 1–6 cells. In addition, ROS can trigger the process of lipid peroxidation in the lipid membrane, and cause damage to DNA by propagating a chain reaction [40]. The hydroxyl radicals and nitric oxide of ROS can oxidize the bases of DNA and cause various types of DNA damage [41]. Many previous studies have shown that DON causes DNA fragmentation leading to cell death and apoptosis [42,43,44,45]. However, there is limited direct evidence suggesting the role of ROS in DNA damage, and some studies report that DON might induce direct lesions to DNA. 8-OHdG is typical evidence or factor of DNA damage caused by ROS. We found that DON exposure caused excessive 8-OHdG levels in Hepa 1–6 cells, which indicated that DON-induced oxidative stress might trigger DNA damage. Therefore, we use the antioxidant enzyme/DNA repair system as an index to indirectly reflect the degree of liver damage. Otherwise, we also found that the antioxidant enzyme activity of the DON group showed an upward pattern compared to the control group, although the difference was not statistically significant, which contradicted the conclusions of previous studies. We found that DON administration for 30 days did not reduce the antioxidant enzyme activity in the liver, which is inconsistent with the conclusions of previous studies. For example, after a 14-day DON intervention (28 mg/kg body weight/day), GSH and CAT in the liver of rats decreased significantly [8]. Moreover, feeding mice with food containing mycotoxins caused a decrease in the activity of CAT and SOD in the liver [11]. It indicates that high doses of DON can severely exhaust the antioxidant system in a short period. However, low-dose DON may up-regulate the transcription of the HO-1 gene and maintain the balance of the antioxidant system [46]. Our previous studies have also observed a similar phenomenon, that short-term exposure to DON triggers adaptive expression of HO-1 [23]. 

Next, we edited overexpression/silence of the HO-1 gene to further explore the protective effect of HO-1 in DON-induced liver injury. When exposed to DON for 30 days, HO-1 overexpression effectively activates the antioxidant enzyme/DNA repair system, reverses liver damage, and significantly reduces the production of ROS and 8-OHdG. The silence of HO-1 leads to the breakdown of the antioxidant enzyme/DNA repair system and the worsening of liver damage. Interestingly, when DON was exposed for 90 days, the antioxidant enzyme/DNA repair system in the HO-1^OE^ group was dysregulated. The possible reason may be that the protective abilities of HO-1 cannot withstand the attacking speed on cellular structures measured in nanoseconds (10^–9^ s) due to hydroxyl group (s) in the chemical structure of DON with long-lasting exposure times [47,48]. In addition, glutathione is the main endogenous antioxidant and is also used in metabolic and biochemical reactions such as DNA synthesis and repair [49,50]. The consumption of GSH is related to the regulation of ROS balance by HO-1 [51]. Therefore, excessive consumption of GSH may be another important reason for the imbalance of the antioxidant enzyme/DNA repair system. Besides, we used a cell model to explore more protective pathways of HO-1 in subchronic DON exposure in vitro, such as autophagy, which is related to the pathology of many human diseases [52]. According to previous studies, different drugs can trigger different HO-1 expression patterns to regulate autophagy under various pathological conditions, such as resveratrol, trehalose, CDDO-imidazole, and so on [53,54,55]. Our in vitro experimental results further show that overexpression of HO-1 can maintain autophagy, while the silence of HO-1 inhibits autophagy. The slight autophagy activation in the DON group may be an adaptive expression of self-protection. Therefore, autophagy may be another meaningful way for HO-1 to protect the liver under the DON-induced oxidative damage in liver tissue and cell line. 

## 4. Conclusions

We evaluate the potential adverse effects of subchronic administration of DON given at doses comparable to the daily human consumption and propose the protective mechanisms of HO-1 against long-term exposure to low-dose DON. In conclusion, DON can cause liver tissue damage under sub-chronic exposure conditions, and it may cause toxic effects on the liver by inducing excessive ROS and DNA damage. In this study, we described the potential mechanism of HO-1 to reduce DON-induced liver damage; meanwhile, HO-1 could directly or indirectly alleviate DON-induced liver damage by regulating the antioxidant enzyme system, DNA repair system, and autophagy. We hope our results enrich the theory of hepatotoxicity induced by DON and provide new insights into the protective mechanism of HO-1 on the liver that could support potential direction for future study.

## 5. Materials and Methods

### 5.1. Reagents

DON (12, 13-epoxy-3, 4, 15-trihydroxytrichotec-9-en-8-one, C15H20O6, MW: 296.32, purity ≥ 99%, CAS RN: 51481-10-8) was purchased from Sigma-Aldrich (St. Louis, MO, USA). Hematoxylin and eosin staining kit (C0105), enhanced BCA protein assay kit (P0010), RIPA lysate (P0013B), and PMSF (ST505) were purchased from Beyotime Biotechnology (Shanghai, China). Phosphatase inhibitor cocktail (B15001) and protease inhibitor cocktail (B1400) were purchased from Bimake (Houston, TX, USA). Mini-reduced glutathione (GSH) assay kit (A006-2-1,96T), superoxide dismutase (SOD) assay kit (A001-3, WST-1), and catalase (CAT) assay kit (A007-1-1, Ammonium molybdate method) were purchased from Nanjing Jiancheng Institute of Biological Engineering (Nanjing, China). Cell Counting Kit-8 (CCK-8) was purchased from Dojindo (Shanghai, China). The 4% phosphate-buffered paraformaldehyde (C1101) was purchased from Servicebio technology (Wuhan, China). The fluorescent probe dihydroethidium (DHE) was purchased from Beyotime (Haimen, China). The ELISA kit (8-OHdG) was purchased from Elabscience Biotechnology Co. Ltd. (Wuhan, China).

### 5.2. Animals

8-week-old SPF male C57BL/6J mice, weighing 16-22 g, were purchased from Beijing Charles River Experimental Animal Technology Co., Ltd., and raised in the SPF animal laboratory of Tongji Medical College, Huazhong University of Science and Technology. The control environment temperature is (22 ± 2) ℃, humidity 60%, 12 h circadian rhythm (day and night 08:00-20:00), with free drinking and eating. After 1 week of adaptive feeding, the mice were randomly divided into four groups according to body weight: control group (N = 10); DON group (N = 10); DON + HO-1^OE^ group (N = 10); DON + HO -1 ^shRNA^ group (N = 10) (Figure S1).

In order to construct HO-1 overexpression recombination and HO-1 silencing recombination AAV8 type, the HO-1 gene sequence and shRNA3 sequence were inserted into pHBAAV-CMV-MCS-ZsGreen plasmid and pHBAAV-U6-ZsGreen plasmid, respectively. Detailed primer sequences and operating methods are provided in the supplementary materials (Figure S2). The viral transfection efficiency of the HO-1 gene in mouse liver was verified by Western blot (Figure S3a,b). All viruses are packaged and purified by Shanghai Hanheng Biological Engineering Co., Ltd. The optimal concentrations of HO-1 overexpression and HO-1 silenced recombinant AAV8 were injected into the HO-1^OE^ group and HO-1 ^shRNA^ group mice (100 μL/mouse) from the tail vein. Three weeks after virus transfection, mice in the DON group, HO-1^OE^ group, and HO-1^shRNA^ group received 25 μg/kg bw/day DON by oral gavage for 30 days and 90 days. Each mouse was gavaged according to the standard of 5μL/g/bw, and the mice in the control group were intragastrically administered with the same standard of ultrapure water.

After the experiment, the mice were sacrificed by cervical vertebrae and dissected immediately. The liver samples for hematoxylin-eosin (H&E) staining were fixed in 4% phosphate-buffered paraformaldehyde for 48 h, and the liver samples were embedded in paraffin and cut into 4 μm sections. The remaining liver samples were quick-frozen in liquid nitrogen and stored at −80 °C for subsequent index determination. The Animal Care and Use Institutional Committee of Tongji Medical College of Huazhong University of Science and Technology (IACUC Number: S407) approved all procedures in animal experiments.

### 5.3. Cell Lines

The mouse liver cancer cell line Hepa 1–6 (CRL-1830™) was obtained from the American Type Culture Collection (ATCC, Manassas, VA, USA). To overexpress HO-1 in Hepa 1–6 cells (HO-1^OE^ Hepa 1–6 cells), we inserted the HO-1 (HMOX-1) sequence into the pHBLV-CMVIE-ZsGreen-Puro vector (Shanghai Han Bioengineering Co., Ltd., Shanghai, China). To silence HO-1 in Hepa 1–6 cells (HO-1^shRNA^ Hepa 1–6 cells), we inserted three different shRNAs into the pHBLV-U6-Scramble-ZsGreen-Puro vector (Shanghai Hanheng Biological Engineering Co., Ltd.). Detailed primer sequences and operating methods are provided in the supplementary materials (Figure S2). The viral transfection efficiency of the HO-1 gene in Hepa 1-6 cells was verified by Western blot (Figure S3c).

Wild-type Hepa 1–6 cells, HO-1^OE^ Hepa 1–6, and HO-1^shRNA^ Hepa 1–6 cells were cultured in Dulbecco’s modified Eagle medium (DMEM) (Gibco, 11, 965-084) containing 10% (*v*/*v*) culture fetal bovine serum (FBS) (Hyclone, SH30084.03) and 1% (*v*/*v*) penicillin/streptomycin at 37 °C in a humidified atmosphere of 5% CO2. The above cells were divided into four groups: Hepa 1–6 cell group (control group); Hepa 1–6 cells + DON group (DON group); HO-1^OE^ Hepa 1–6 cells + DON group (HO-1^OE^ Hepa 1- Group 6); HO-1^shRNA^ Hepa 1–6 cells + DON group (HO-1^shRNA^ Hepa 1–6 group). Culture cells of the same or similar generation were transferred to a 100 mm Petri dish (Corning-Costar, USA). After two passages, when the cells reached 80% confluence, the medium was changed to DMEM medium containing 0.6 μM DON and 5% FBS, and the drug was administered for 24 h. Cells were collected for subsequent index measurement.

The cells after DON intervention were collected and resuspended in PBS. Cells were incubated on ice followed by sonication to break the cells using a sonicator (SONICS & MATERIALS, Newtown, CT, USA) at 30% amplitude for 10 s, and repeated three times. The cell supernatant was collected by centrifugation at 3000 rpm for 10 min, and the SOD, CAT, and GSH assays were performed in triplicate.

### 5.4. Determination of Antioxidant Enzyme Contents in Tissues and Cells

Mouse liver samples were prepared with 10% liver homogenate by a high-throughput tissue grinder (Ningbo Xinzhi Biotechnology Co., Ltd. Company, Ningbo, China). The tissue homogenate was centrifuged at 2500 r for 10 min, and the supernatant taken. The protein is quantified by the bicinchoninic acid (BCA) method. A multi-functional microplate reader measured the optical density (OD) values of SOD, CAT, and GSH at 450 nm, 405 nm, and 405 nm (Bio-Tek, Winooski, VT, USA). The instructions on the kit of Nanjing Jiancheng Company was followed.

### 5.5. Determination of 8-OHdG Levels

The content of DNA adducts in Hepa 1–6 cells induced by DON was detected by 8-OHdG ELISA kit (Elabscience Biotechnology Co. Ltd., Wuhan, China). Cells in DON group, HO-1^OE^ Hepa 1–6 group, and HO-1^shRNA^ Hepa 1–6 group were treated with 0.6 μM DON for 24 h. After the incubation, the cells of each group were collected, sonicated to disrupt the cells, centrifuged at 1000× *g* for 20 min to remove impurities and cell debris, and the supernatant was taken for detection. Then, referring to the kit instructions, using a microplate reader (Bio-Tek, Winooski, VT, USA), optical density (OD) value of each well at a wavelength of 450 nm was measured.

### 5.6. Western Blot Analysis

Western blot analysis is based on our previous research protocol with a slight modification [56]. Ice-cold RIPA buffer containing 1% phenylmethanesulfonyl fluoride (PMSF) was used to lyse cells or tissue samples by ultrasound on ice thoroughly. The proteins are separated by a 12% acrylamide denaturing gel size, and transferred to a 0.45 μm nitrocellulose (NC) membrane (Millipore, HATF00010), and blocked with 5% skimmed milk in TBST. Then The sample was incubated with the corresponding primary antibody overnight at 4 °C, and then incubated with the horseradish peroxidase-conjugated secondary antibody for 1 h. NC membrane sprinkled with ECL coloring solution was placed in the gel imaging analyzer (Syngene, Cambridge, UK), and GeneSnap (Syngene, Cambridge, UK) and GeneTools (Syngene, GeneTools 4.0, Cambridge, UK) were used to collect and analyze the images. ImageJ software is used for semi-quantitative analysis of the grayscale of protein bands. Information for antibodies is included in Table S1.

### 5.7. Cellular ROS Measurement

The fluorescent probe dihydroethidine (DHE, Beyotime, Haimen, China) was used to detect the formation of ROS in Hepa 1–6 cells induced by DON. Dihydroethidium reacts with ROS in living cells to produce ethidium by dehydrogenation, which can combine with RNA or DNA to produce red fluorescence. After DON administration, the cells were incubated with DHE (5 μM) at 37 °C in the dark for 30 min, and then washed with PBS to remove excess probes. Fluorescence was obtained at 535 nm by an inverted fluorescence microscope (Olympus Corporation, Tokyo, Japan), and images were collected and analyzed by ImageJ software.

### 5.8. Statistical Analysis

Statistical software SPSS 20.0 (SPSS Inc., Chicago, IL, USA) was used for data processing and statistical analysis. All experimental results were expressed as mean ± standard deviation (*x* ± SD). One-way analysis of variance (ANOVA) was used for comparison among multiple groups. Significance was defined at * *p* < 0.05, ** *p* < 0.01.

## Data Availability

The data that support the findings of this study are available from the corresponding author upon reasonable request.

## Supplementary Materials

The following are available online at https://www.mdpi.com/article/10.3390/toxins13100732/s1, Table S1: List of Antibodies for WB., Figure S1. The grouping and treatment of experimental animals., Figure S2. Virus transfection vector. (a) Lentiviral vector. (b) Adeno-associated virus vector., Figure S3. Validation of the efficiency of AAV8 transfecting the HO-1 gene. HO-1 overex-pres-sion/silencing efficiency in mouse liver after 30 days (a) and 90 days (b) of DON admin-istration. (c) HO-1 overexpression/silencing efficiency in Hepa 1-6. Data are expressed as mean ± SD; “*” means *p* < 0.05; “**” means *p* < 0.01.

## Abbreviations

DON	Deoxynivalenol;
HO-1	Heme oxygenase-1;
CAT	Catalase;
GSH	Glutathione;
8-OHdG	8-hydroxy-2 deoxyguanosine;
ROS	Reactive oxygen species;
SOD	Superoxide dismutase;
ERCC1	Excision repair cross-complementation group 1;
XPC	Xeroderma pigmentosum complementation group C;
OE	Overexpression;
shRNA	Short hairpin Ribonucleic Acid.
