# Supplementary material for: The Protective Effect of Heme Oxygenase-1 on Liver Injury Caused by DON-Induced Oxidative Stress and Cytotoxicity
Source: Toxins (Basel). 2021 Oct 17;13(10):732. doi: 10.3390/toxins13100732 (PMC8541417; doi:10.3390/toxins13100732)
Supplement: Supplementary file 1 [file toxins-13-00732-s001.zip › toxins-1362283-supplementary.pdf]

Supplementary Materials: Biofilm-Forming Methicillin-Resistant *Staphylococcus aureus* Survive in Kupffer Cells and Exhibit High Virulence in Mice

Zitong Meng, Liangliang Wang, Yuxiao Liao, Zhao Peng, Dan Li, Xiaolei Zhou, Shuang Liu, Yanmei Li, Andreas K. Nüssler, Liegang Liu, Liping Hao and Wei Yang

Table S1. List of Antibodies for WB.

No.	Antibody	Manufacturer	applied dilution times	Catalog Number
1	LC3B	Cell Signaling Technology	1000	#3868
2	SQSTM1/p62	Cell Signaling Technology	1000	#5114
3	Atg5	Cell Signaling Technology	1000	#12994
4	ATG12	Cell Signaling Technology	1000	#4180
5	Beclin-1	Cell Signaling Technology	1000	#3495
6	GAPDH	Cell Signaling Technology	2000	#97166
7	XPC	Cell Signaling Technology	1000	#14768
8	ERCC1	Cell Signaling Technology	1000	#12345
9	β-actin	Cell Signaling Technology	2000	#3700
10	HO-1	Abcam	1000	#ab13248
11	(HRP)-linked anti-rabbit IgG	Cell Signaling Technology	10000	#7074
12	(HRP)-linked anti-mouse IgG	Cell Signaling Technology	10000	#7076

Supplementary methods

1. In vitro transfection and construction of stable HO-1 overexpression and silence cell lines

For overexpression of HO-1 in Hepa 1–6 cells (HO-1^{OE} Hepa 1–6 cells), the HMOX-1 sequence was inserted into the pHBLV-CMVIE-ZsGreen-Puro vector (Fig. S2a) (Hanbio Technology Co. Ltd, Shanghai, China). Primers include: m-HMOX1-Eco/Eco-F primer, and m-HMOX1-Eco/Eco-R primer. For silence of HO-1 in Hepa 1–6 cells (HO-1shRNA Hepa 1–6 cells), three different shRNAs were inserted into the pHBLV-U6-Scramble-ZsGreen-Puro vector (Fig. S2a) (Hanbio). Stable HO-1 overexpression and silence cell lines were harvested for quantitative polymerase chain reaction (qPCR) after 14 h transfection at 37 °C and were selected using 1 μg/mL puromycin before DON administration.

2. AAV8-mediated overexpression and silence of HO-1 in the liver

For construction of HO-1 overexpression recombinant adeno-associated virus serotype 8 (HO-1OE AAV8), mouse HMOX-1 gene sequence was packaged into

pHBAAV-CMV-MCS-ZsGreen vector (Fig. S2b). Primers include: m-HMOX1-Bam-kpn-F primer, and m-HMOX1-Bam-kpn-R primer. For construction of HO-1 silence recombinant AAV8 (HO-1shRNA AAV8) viral vectors, shRNA3 was packaged into pHBAAV-U6-ZsGreen vector (Fig. S2b). All recombinant AAV8 were purified by HanBio Technology Co. Ltd (Shanghai, China). Accordingly, based on a separate pre-experiment of determining an optimal virus injection dose, mice were injected HO-1OE or HO-1shRNA AAV8 (1 × 1012 viral particles, 100 μL/mouse) via tail vein after 1-week acclimation and then housed for 4 weeks (Fig.S1). Liver samples were collected after DON administration and western blot assays were used to examine the HO-1 expression in liver.

3. Verification of the efficiency of HO-1 overexpression and silencing

During low-dose DON exposure, the viral transfection efficiency of the HO-1 gene in mouse liver and Hepa 1-6 cells was verified by Western blot. Compared to the control group and the DON group, the expression level of HO-1 in HO-1OE group was signifi-cantly increased, and in HO-1shRNA group was significantly reduced, regardless of mouse liver or Hepa 1-6 cells (Fig. 3a-c). These results indicate that HO-1 overexpres-sion/silencing cells or animal models have been successfully constructed.

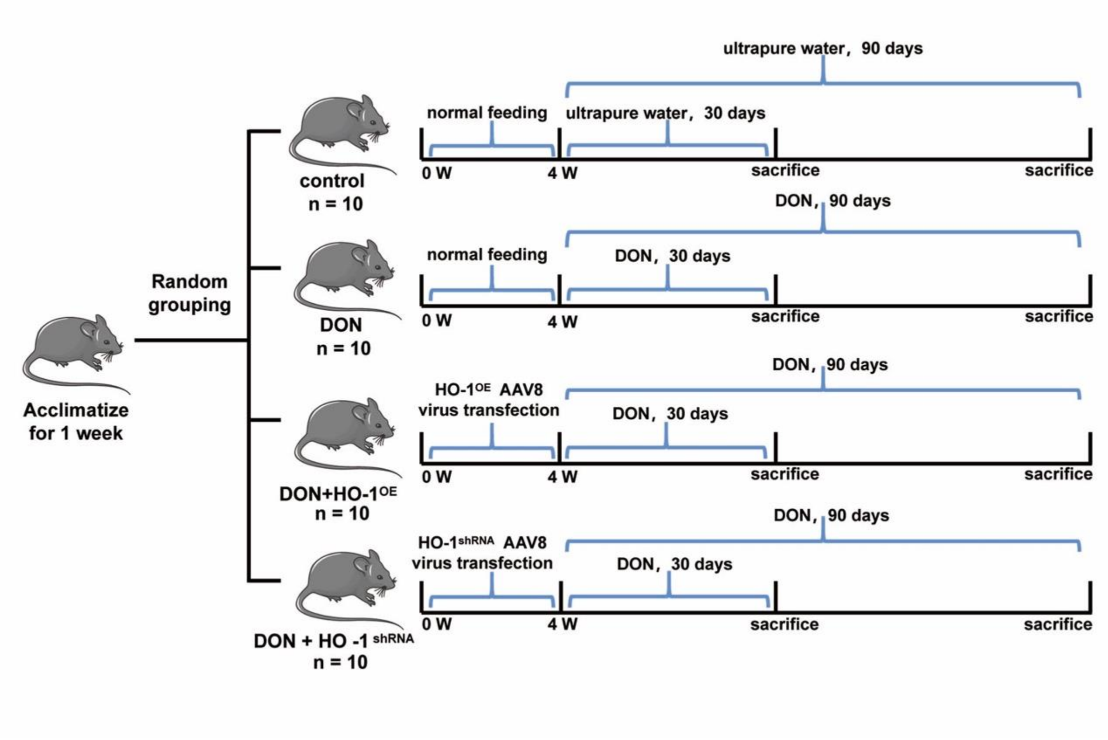

Figure S1. The grouping and treatment of experimental animals.

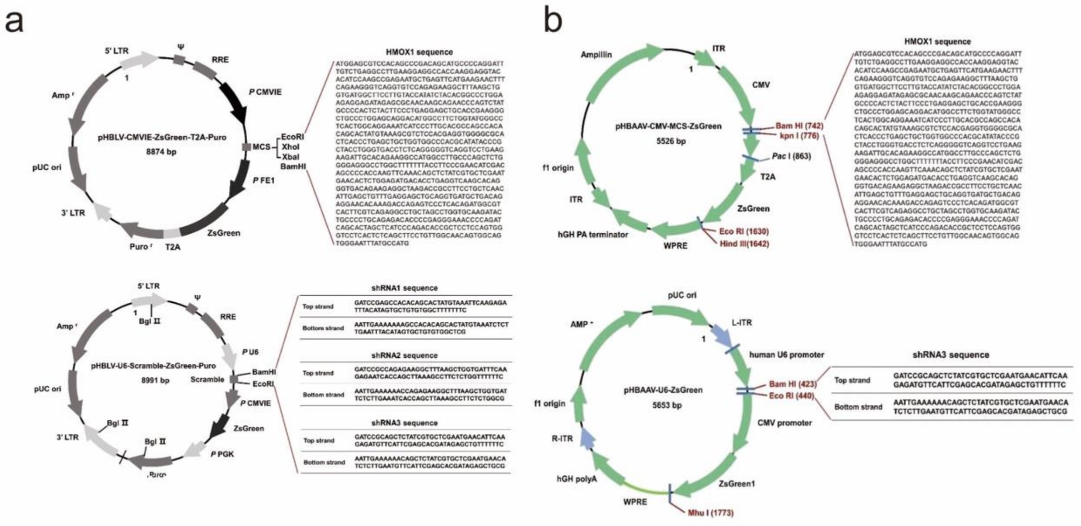

Figure S2. Virus transfection vector. (a) Lentiviral vector. (b) Adeno-associated virus vector.

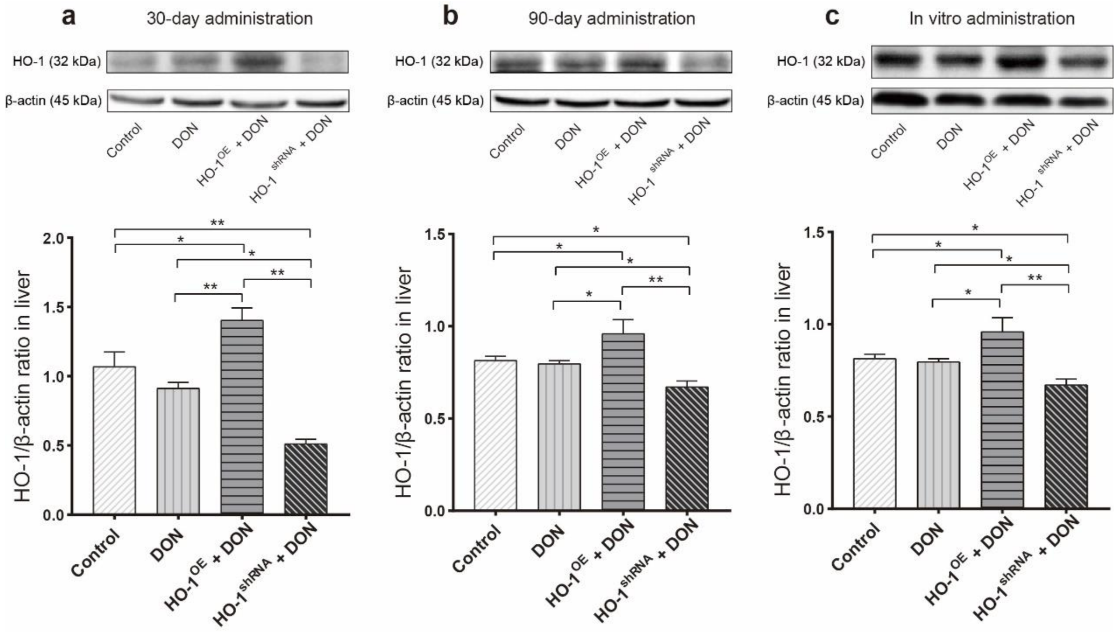

Figure S3. Validation of the efficiency of AAV8 transfecting the HO-1 gene. HO-1 overexpres-sion/silencing efficiency in mouse liver after 30 days (**a**) and 90 days (**b**) of DON administration. (**c**) HO-1 overexpression/silencing efficiency in Hepa 1-6. Data are expressed as mean ± SD; "*" means P <0.05; "**" means P <0.01.
